# The analgesic efficacy of ultrasound-guided transversus abdominis plane (TAP) block combined with oral multimodal analgesia in comparison with oral multimodal analgesia after caesarean delivery: a randomized controlled trial

**DOI:** 10.1186/s12871-020-01223-3

**Published:** 2021-01-07

**Authors:** Yang Yu, Shenshan Gao, Vivian Man-ying Yuen, Siu-Wai Choi, Xuebing Xu

**Affiliations:** 1 Department of Anaesthesiology, University of Hong Kong - Shenzhen Hospital, Shenzen, Guangdong China; 2 Department of Anaesthesiology and Perioperative Medicine, Hong Kong Children’s Hospital, Pittsburgh, Hong Kong; 3 Department of Oromaxillofacial Surgery, University of Hong Kong, Pok Fu Lam, Hong Kong

**Keywords:** Transversus abdominis plane block, Oral analgesics, Caesarean deliver

## Abstract

**Background:**

The transversus abdominis plane (TAP) block is used increasingly in parturients after caesarean delivery. This is a randomized controlled trial to evaluate the effectiveness of bilateral single-shot of TAP blocks in patients who received multimodal oral analgesia for postoperative pain relief.

**Methods:**

Parturients who were scheduled for elective caesarean delivery under spinal anaesthesia were recruited and randomized to receive bilateral single-shot of TAP blocks or placebo in addition to multimodal oral analgesia which consisted of regular tramadol, celecoxib and paracetamol, with oral oxycodone used as a rescue for breakthrough pain. Only parturients in the TAP group would receive the TAP blocks with an injection of 15 ml (0.25%) ropivacaine on each side under aseptic techniques. All the parturients were evaluated for pain or related complications in the first 24 h after surgery. The primary outcome is the percentage of parturients who required oxycodone as a rescue analgesia.

**Results:**

Eighty and 79 parturients were allocated to the TAP and placebo group respectively. Nine out of 79 (11.4%) parturients in the TAP group and 15 out of 73 (20.5%) parturients in the placebo group required oxycodone for breakthrough pain, *P* = 0.122.

**Conclusions:**

Bilateral single-shot of TAP blocks confer little additional benefit when a multimodal oral analgesic regimen is used for pain control after caesarean section under spinal anaesthesia.

**Trial registration:**

Clinical Trial Registry of China (http://www.chictr.org.cn) identifier: ChiCTR-INR-16010130, retrospectively registered on Dec 12, 2016.

**Supplementary Information:**

The online version contains supplementary material available at 10.1186/s12871-020-01223-3.

## Background

With the availability of ultrasound machines, transversus abdominis plane (TAP) block may be easily performed after a caesarean section for postoperative analgesia. It was shown to be effective to relieve pain and reduced intravenous morphine consumption in patients who used patient-controlled intravenous morphine analgesia for post-caesarean pain relief [1]. Previous systemic reviews and meta-analyses have revealed that it is only effective after caesarean delivery and provides effective analgesia when spinal morphine is not used [2, 3], therefore TAP blocks offer few additional benefits when patients have intrathecal morphine administered. Although intrathecal morphine is the best option for post caesarean pain relief [4], preservative free morphine is not available in our center and most parts of China. Our delivery rate increased from 200 per year in 2014 to 7000 per year to date, with a 20–30% cesarean delivery rate. The routine post-caesarean analgesia in our center consists of multimodal oral analgesia including regular tramadol (SR 100 mg BID for 2 days), celecoxib (200 mg BID for 3 days) and paracetamol (1000 mg QID for 4 days), with oral oxycodone (10 mg PRN) as rescue pain relief. Our quality assurance exercise revealed satisfactory pain control with this regime, and the proportion of patients who required oxycodone rescue range from 15 to 20% and less than 0.5% of patients would need intravenous morphine as rescue analgesia. Since TAP blocks have been shown to significantly reduce intravenous morphine consumption in the first 24 h after a caesarean section, we conducted this double-blind randomized controlled trial to test the hypothesis that TAP blocks would further improve post-caesarean analgesia in patients who have multimodal oral analgesia.

## Methods

The study was carried out in a tertiary care public hospital in Shenzhen, China. It was approved by the Hospital Institutional Review Board (szkcw201628) and registered with the Clinical Trial Registry of China (ChCTR-INR-16010130). After patient informed consent was obtained, the parturients scheduled for elective caesarean delivery under spinal anaesthesia were enrolled in the study. Spinal anesthesia was performed in a lateral position at the L2/3 or L3/4 interspace with 0.5% hyperbaric bupivacaine 10 mg (2 ml) combined with fentanyl 15 μg (0.3 ml) in both groups. Inclusion criteria were American Society of Anaesthesiologists physical status I or II parturients, scheduled for elective caesarean delivery under spinal anesthesia. Exclusion criteria included a body mass index of more than 35 kg/m^2^, major systemic disease, chronic pain disorders, neurological disorders, abuse of drugs or alcohol, and allergies to any medication included in the study protocol and inability to comprehend or use the visual rating pain scoring system.

An independent statistician prepared a randomization list while block of group allocation was kept in concealed opaque envelops. Attending anaesthetists would disclose group assignments at the start of surgery, and prepare for the TAP blocks if necessary. In order to ensure the recruited parturients who had spinal anaesthesia were blind from group allocation and to avoid sham block, the surgical drape which blocks the parturient’s view of her surgical site was kept after surgery. After a wound was covered with dressing, an ultrasound-guided bilateral single-shot of TAP blocks was performed by the attending anaesthetists who are experienced with this technique before conducting the investigation. A linear 13- to 6-MHz ultrasound probe (Sonosite™, Bothell, Washington) was placed transversely on the anterolateral abdominal wall between the iliac crest and costal margin. The three layers of muscles - the external oblique, the internal oblique, and the transversus abdominis - were identified. Only parturients who were allocated to have TAP blocks would receive an injection of local anaesthesia under aseptic techniques. A 22-gauge, 90-mm SonoPlex Stim needle (Pajunk Medizintechnik, Geisingen, Germany), attached with flexible tubing to a syringe filled with 0.9% normal saline, was introduced through the skin anteriorly in the plane of the ultrasound beam, and advanced into the fascial plane between the internal oblique muscle and transversus abdominis muscles. Ropivacaine(Naropin, Astrazeneca AB. Sweden)15 ml (0.25%) was injected on each side of the abdominal wall for the TAP blocks. A local anaesthetic solution was injected in 5 ml increments after aspiration. After each 5 ml bolus, patients were monitored for an increase in heart rate or signs of local anesthetic toxicity such as tinnitus, perioral numbness, metallic taste in mouth, slurring of speech, and mental status changes. Since the parturients who had effective spinal block would not feel the needle injection of TAP blocks, it was possible to blind the participants from group allocation as an ultrasound scan would be performed even if TAP blocks were not administered.

All recruited patients had intravenous parecoxib 40 mg before the end of the operation. They were also prescribed multimodal oral analgesics postoperatively, and this included slow release tramadol 100 mg twice a day for the first two days, celecoxib 200 mg twice a day for three days, and paracetamol 1000 mg four times a day for four days. Oxycodone 10 mg for once as PRN was prescribed and used when necessary.

Participants were given instructions to fill in a survey for postoperative pain control. The numeric rating scale (NRS) for pain and satisfaction scale was explained. NRS consists of scores of 0 to 10, where 0 equal to no pain and 10 equal to the worst pain. A scale of 5 was used as a satisfaction score, with 1 equal to “very unsatisfactory” and 5 equal to “very satisfactory” with pain relief. The participants were asked to record the NRS at rest, with movement, and with uterine massage and the satisfaction score at 2 h, 4 h, 6 h and 12 h after completion of surgery. In addition, they were asked to record if there was an episode of nausea and vomiting. The parturients would also record if oxycodone or uterotonic was used in the first 24 h. Moreover, they would also record if there was any instance of pain when the NRS score was greater than 6 in the first 24 h. The survey was collected by the pain nurse during follow up on day 1 after the operation.

The primary outcome is the percentage of parturients who required oxycodone as rescue analgesia. Secondary outcomes include the NRS at rest, NRS with movement and NRS during uterine massage, patient satisfaction score, the percentage of parturients who experienced pain with NRS > 6 during the first 24 h after surgery, and the incidence of nausea and vomiting. According to our record of routine postoperative visits, approximately 20% of our patients required oxycodone as rescue pain relief. If TAP blocks were to decrease the requirement of oxycodone from 20 to 5%, 73 parturients per group would be required for 80% power with a 5% type 1 error.

Parametric primary and secondary outcomes are presented as the mean (SD) or number (percentage) and were compared by t-test or Chi-square test. Non-parametric data are presented as the median (IQR [range]) and compared by the Mann-Whitney U test. The area under the curve (AUC) for pain scores and satisfaction scores was derived using the trapezoidal rule. The mean AUC of pain scores and satisfaction scores were presented as the mean (SD) and compared using a t-test. A *P* value < 0.05 was considered significant. Data were analysed using SPSS (version 21.0; SPSS Inc., Chicago, IL, USA).

## Results

**Fig. 1:**
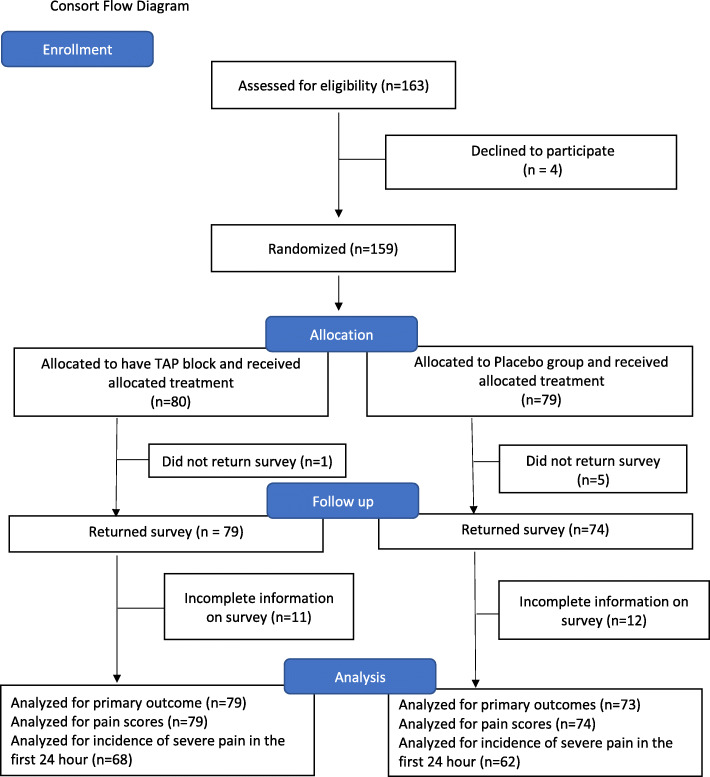
Consort flow diagram. Patients were excluded because of incompleting information

**Fig. 2:**
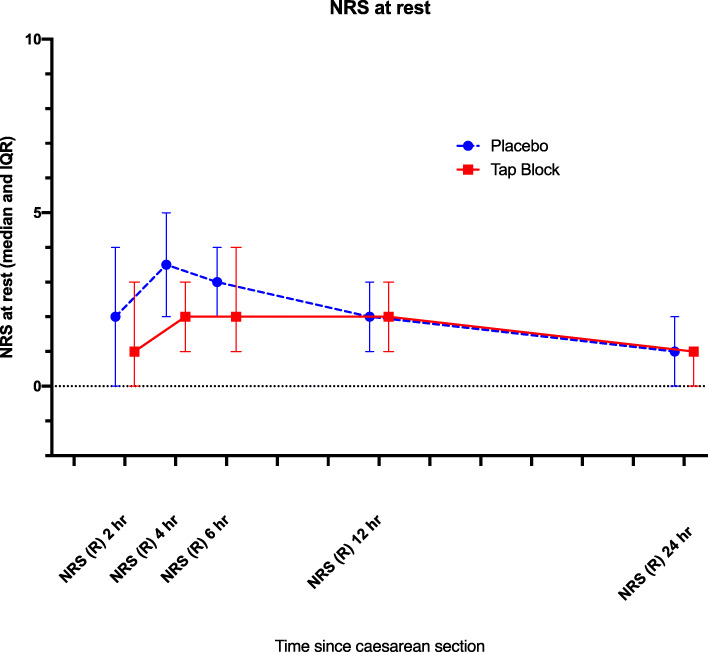
NRS at rest. The median (IQR) pain score in numeric rating scale (NRS) at rest in the placebo group ( ) and the TAP group ( ) over the first 24 h after Caesarean section

**Fig. 3:**
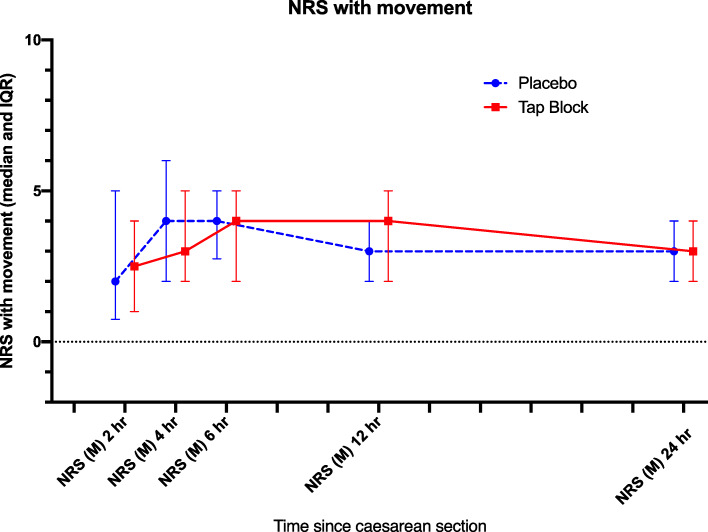
NRS with movement. The median (IQR) pain score in numeric rating scale (NRS) with movement in the placebo group ( ) and the TAP group ( ) over the first 24 h after Caesarean section

**Fig. 4:**
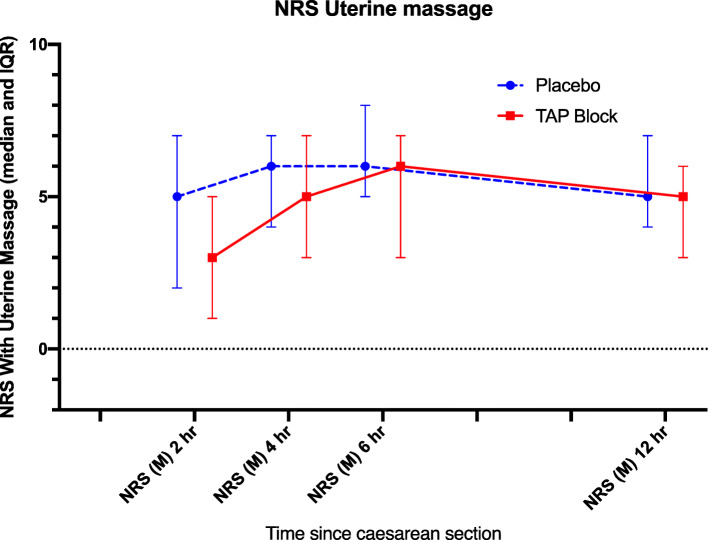
NRS Uterine massage. The median (IQR) pain score in numeric rating scale (NRS) with uterine massage in the placebo group ( ) and the TAP group ( ) over the first 12 h after Caesarean section

One hundred and sixty-three patients were approached, and 159 patients consented to take part in this study between April and September in 2016. Eighty parturients received TAP blocks and 79 were allocated to the placebo group (Fig. 1). One patient from the TAP group and five patients from the placebo group were excluded because either the patients lost the record sheets or forgot to record any data. As a result, a primary outcome was available from 79 and 73 parturients in TAP and placebo group respectively. In all patients in the TAP group, the transversus abdominis neurofascial plane was localized by ultrasound easily, and the block was performed without complication. Demographic information is presented in Table 1. Nine out of 79 (11.4%) and 15 out of 73 (20.5%) parturients required oxycodone for breakthrough pain (*P* = 0.122, Table 3). The AUC of NRS for pain at rest (Fig. 2) and during movement (Fig. 3) for the first 24 h was not different between the two groups (*P* = 0.87 and *P* = 0.95 respectively, Table 2). The AUC of NRS for pain during uterine massage (Fig. 4) for the first 12 h was also not different between the two groups (*P* = 0.66, Table 2). The AUC of the patient satisfaction score for the first 12 h was not different between the two groups (*P* = 0.58, Table 2). There was no difference in the incidence of nausea and vomiting between the two groups and a similar proportion of patients who required a uterotonic for the controlling of bleeding (Table 3). Sixty-eight patients in the TAP group and 62 patients in the placebo group were analyzed for the patients who experienced severe pain (with NRS > 6 at any time point) because some patients failed to return the postop pain survey. Thirty-two of 68 (47.1%) patients in the TAP group experienced severe pain (with NRS > 6 at any time point) versus 39 of 62(62.9%) patients in the placebo group (*P* = 0.07) during the first 24 h post-operatively (Table 3).

**Table 1:** Subjects’ characteristics

	TAP(*n* = 79)	Placebo(*n* = 74)
Age (year)	32.4 ± 3.5	32.5 ± 4.0
Body Weight (kg)	66.4 ± 6.9	67.0 ± 7.1
Body Height (cm)	159.5 ± 4.8	160.2 ± 5.0
BMI	26.1 ± 2.4	26.1 ± 2.6
Gestation	39 [37–39]	39 [37–39]
Gravida	2 [1–3]	2 [1–3]
Parity 0:01:02	42:36:01 (53.2%: 45.6%: 1.3%)	25:46:03 (33.8%: 62.2%: 4.1%)
Blood Loss	200 [200–300]	200 [200–300]
Operation Duration (min)	58.8 ± 14.4	62.3 ± 17.4

*TAP* transversus abdominis plane, *BMI* = body mass index

Values in mean ± SD, median [IQR] or *n*(%)

**Table 2:** AUC of numeric rating scale (NRS) of post-caesarean delivery pain

	TAP(*n* = 79)	Placebo(n = 74)	*p* value
AUC NRS(R) during 2–24 h	20.8 (7.1)	22.4 (7.1)	0.87
AUC NRS(M) during 2–24 h	37.5 (8.0)	38.2 (7.8)	0.95
AUC NRS(U) during 2–12 h	24.3 (5.3)	27.6 (5.3)	0.66
AUC Satisfaction Scores during 2–12 h	9.8 (2.1)	11.6 (2.3)	0.58

AUC of numeric rating scale (NRS) over the first 24 h post-caesarean delivery at rest (NRS (R)), with movement (NRS (M)). The NRS over the first 12 h with uterine massage (NRS (U)) and AUC of satisfaction score over the initial 12 h post-caesarean delivery. *NRS* numeric rating scale*, AUC* area under the curve; Values are in mean (SE)

**Table 3:** The incidence of nausea, vomiting, use of uterotonic, oxycodone and NRS > 6 at any instance during the first 24 h

	TAP (*n* = 79)	Placebo(*n* = 73)	*p* value
Nausea	16 (20.30%)	8 (11%)	0.108
Vomit	5 (6.30%)	8 (11%)	0.32
Uterotonic	51 (64.60%)	46 (63.00%)	0.843
Oxycodone	9 (11.40%)	15 (20.50%)	0.122
NRS > 6 at any instance during the first 24 h	(*n* = 68) 32 (47.10%)	(*n* = 62) 39 (62.90%)	0.07

*TAP* transversus abdominis plane*, NRS* numeric rating scale

Values are *n* (%

## Discussion

Our study demonstrated that TAP blocks were not associated with reducing the use of rescue oxycodone, and we revealed in this study that bilateral single-shot of TAP blocks conferred little additional benefit when a multimodal oral analgesic regimen inclusive of tramadol, celecoxib and paracetamol was used.

With the advancement of ultrasound technology, TAP blocks have become technically easier and safer to perform [5]. Ultrasound-guided nerve blocks offer the advantage of real-time imaging of the needle trajectory and injection spread, which may improve both safety and block effectiveness. Yet, the analgesic efficacy of TAP blocks for caesarean delivery remains controversial. It was associated with effective analgesia in patients after caesarean delivery when spinal morphine was not used [2, 3].Additionally, TAP blocks had been shown to be inferior to spinal morphine for post-caesarean delivery pain [4, 6], and they were ineffective when spinal morphine was used [3, 7, 8]. In this case, this technique would be up to the individual anaesthesiologist or department to decide whether it is a cost-effective and worthwhile additional procedure in their own settings.

The advantage of multimodal oral analgesia is the ease of drug administration without device request. This would facilitate early mobilization after surgery. Recently the U.S. Food and Drug Administration has issued a warning to mothers that breastfeeding is not recommended when taking tramadol due to the risk of serious adverse reactions in breastfed infants [9]. In a recent review on tramadol use in breastfeeding mothers and new-borns, it was revealed that there were no reported deaths of breastfed newborns in association with maternal tramadol use [10].

Since patients and pain nurses who performed the postoperative visits were blind to group assignments, this allowed us to evaluate the effectiveness of TAP blocks without bias. Moreover, all anaesthetists who performed the block were experienced in doing TAP blocks. After all, with the use of ultrasound, TAP blocks were easily performed. Since the postoperative pain score was recorded by the patients themselves before our routine postoperative pain round, this would also further reduce the possibility of bias regarding pain assessment. However, it was also difficult to exclude failed TAP blocks, which served as a cause for analgesia inefficacy due to the fact that residual sensory block from spinal maintained.

## Conclusions

In conclusion, this double blinded randomized controlled trial revealed that bilateral single-shot TAP blocks confer little additional benefit when a multimodal oral analgesic regimen is used for post caesarean section. It would be interesting to investigate if intrathecal morphine would confer any additional benefit when a multimodal oral analgesic regimen is used for post caesarean section pain relief.

## Supplementary Information

**Additional file 1.** CONSORT Checklist

## Data Availability

The datasets used and analyzed during the current study are available. from the corresponding author for reasonable request. The datasets used are also available from Clinical Trial Registry of China. (http://www.chictr.org.cn/hvshowproject.aspx?id=11718)

## Abbreviations

TAP: Transversus abdominis plane
NRS: Numeric rating scale
AUC: Area under the curve
